# A Rare Case of a Mesenteric Cyst

**DOI:** 10.7759/cureus.32015

**Published:** 2022-11-29

**Authors:** Anurag Bhattacharjee, Varun Kulkarni, Yashwant Lamture, Tushar Nagtode, Harshal Ramteke

**Affiliations:** 1 Department of General Surgery, Jawaharlal Nehru Medical College, Datta Meghe Institute of Medical Sciences, Wardha, IND

**Keywords:** mesenteric cyst, exploratory laparotomy, partial drainage, cyst, mesentery

## Abstract

A mesenteric cyst is an uncommon ailment that can affect practically any abdominal quadrant in its presentation. They may turn up as an accidental discovery. Although there are a number of hypotheses explaining the genesis of these cysts, the exact etiology is unknown. A 70-year-old female patient came to see us complaining of abdominal pain for a month and had trouble passing stools for 15 days. Contrast-enhanced computed tomography was done for the patient, which revealed a heterogeneously enhancing mass lesion in the abdominal cavity. The patient was then taken for an exploratory laparotomy procedure. To make the procedure thorough and easy, intraoperative partial drainage of the cyst fluid was carried out. We were able to observe the margins of the mesenteric cyst more easily as the partial drainage was carried out. The partial drainage decreased the volume and size of the mesenteric cyst, reducing the pressure effect on the surrounding structures and allowing easy mobilization of the intraabdominal structures during intraoperative examinations. The partial drainage of the cystic fluid also made the dissection process safer. After releasing all adhesions, the cyst was delivered outside and sent for histopathological analysis. The histopathological reports confirmed it to be a mesenteric cyst. The aim of this article is to educate the readers and to make fellow surgeons well aware of this condition. This will not only help fellow clinicians in better diagnosis and treatment but also help in the reduction of the overall burden of the healthcare society by reducing mortality and morbidity.

## Introduction

Cysts in the small bowel or colon's mesentery are known as mesenteric cysts [1,2]. They are rare, benign intraabdominal tumors that affect people of all ages, with a female-to-male ratio of 2:1 and documented incidences of 1/100,000 in adults and 1/20,000 in children [3]. Because of their size and location, cysts are frequently asymptomatic and are only found by accident during regular imaging. Acute or chronic nonspecific abdominal discomfort (55%-81%), a palpable mass (44%-61%), abdominal distension (17%-61%), as well as nausea and vomiting (45%), constipation (27%), and diarrhea (6%) are the most common symptoms [4]. Chylolymphatic, simple (mesothelial), heterogeneous, urogenital remnant, and dermoid (teratomatous cysts) mesentery cysts are the five different types [4]. The likelihood of recurrence following complete excision using laparoscopic or open techniques is minimal. Although there are a number of theories on how these cysts grow, the exact cause of their growth is unknown [5]. In such circumstances, the preferred course of treatment is the complete surgical removal of the cyst. Early diagnosis is challenging because this ailment is quite uncommon and includes symptoms that are generally vague. Early preoperative diagnosis of these mesenteric cysts is crucial in deciding the plan of management because the many complications related to late diagnosis can lead to poor surgical outcomes. The aim of this article is to educate the readers and to make fellow surgeons well aware of this condition. This will not only help fellow clinicians in better diagnosis and treatment but also help in the reduction of the overall burden of the healthcare society by reducing mortality and morbidity.

## Case presentation

**Figure 1:**
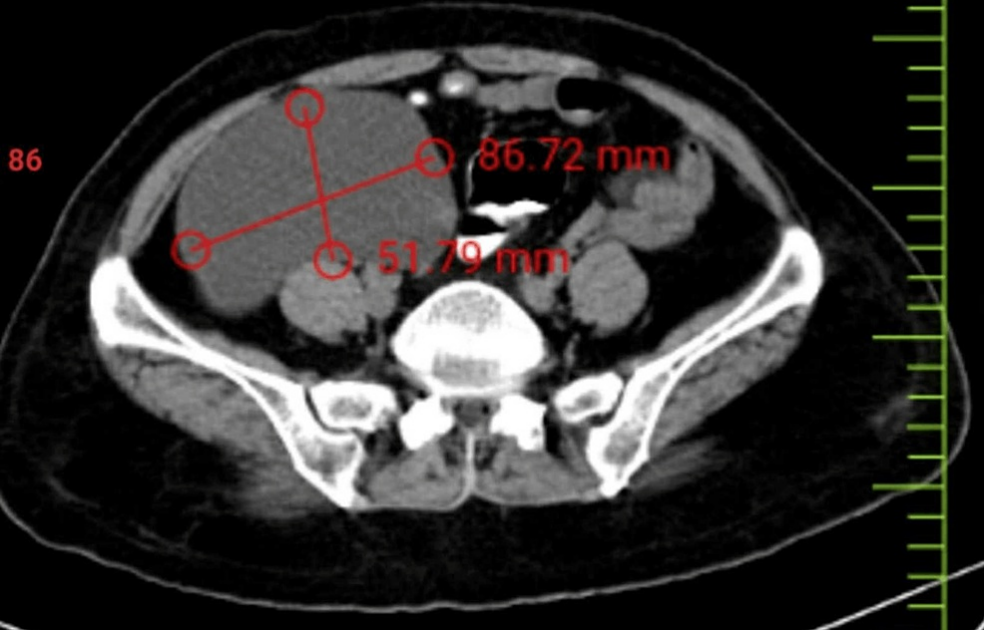
CT scan showing the mesenteric cyst

A 70-year-old female patient came to our emergency department complaining of abdominal pain for a month and having trouble passing stool for 15 days. Clinical evaluation revealed that the patient was in good general condition and that there were no abnormal findings in the systemic examination. However, during the abdominal examination, a mass was palpable in the right iliac fossa region, and it moved perpendicular to the direction in which the mesentery is attached but was constrained in the direction parallel to it. There was a cystic swelling in the lower abdomen after an ultrasonogram (USG) of the abdomen and pelvis. A contrast-enhanced computed tomography scan of the abdomen and pelvis revealed a large, well-defined exophytic, heterogeneously enhancing, solid-cystic mixed-density mass lesion measuring 8.7x8.5x8 cm in the central mesenteric region that abutted the inferior wall of the third part of the duodenum and the proximal jejunum. The mass also abutted the right ureter and inferior vena cava (Figure 1).

**Figure 2:**
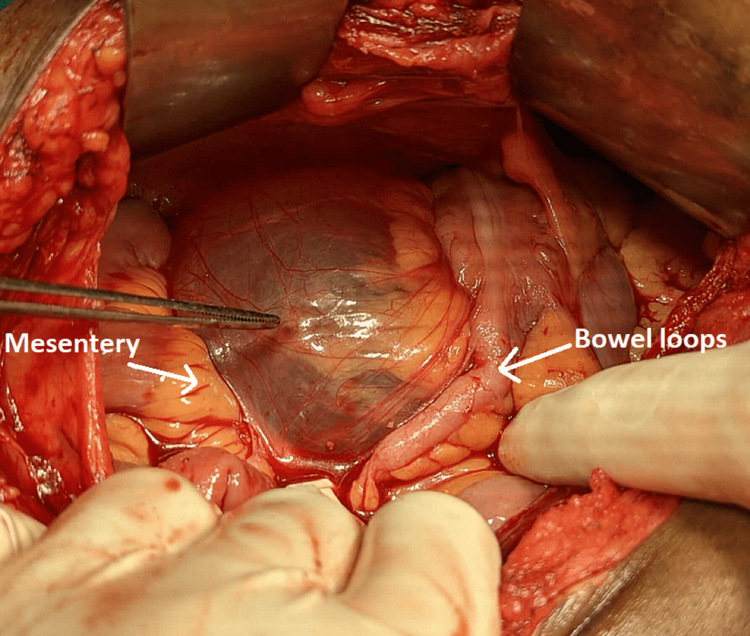
Cyst in situ

After all routine investigations, the patient was taken for surgery. Intraoperatively, cystic growth was seen (Figure 2). In our case, significant adhesions were removed by blunt dissection after the initial examination revealed a cystic mass of 8.5x8.0 cm arising from the mesentery lying beneath the bowel loops.

**Figure 3:**
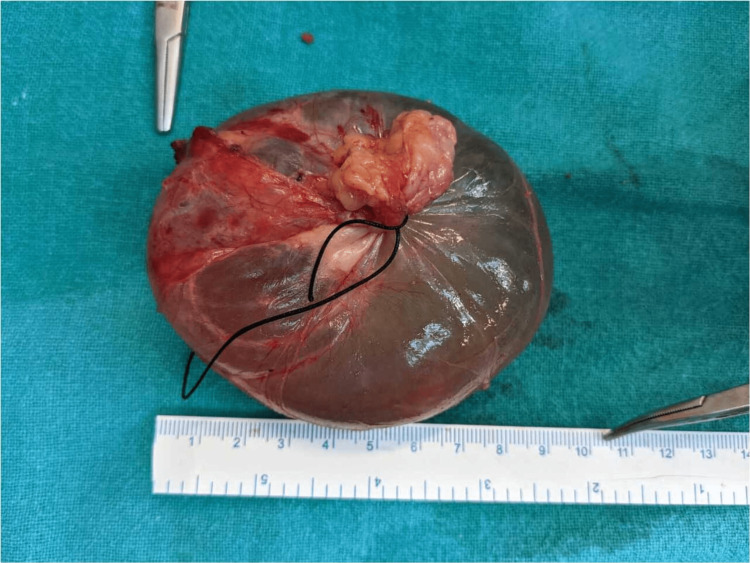
Excised cyst

**Figure 4:**
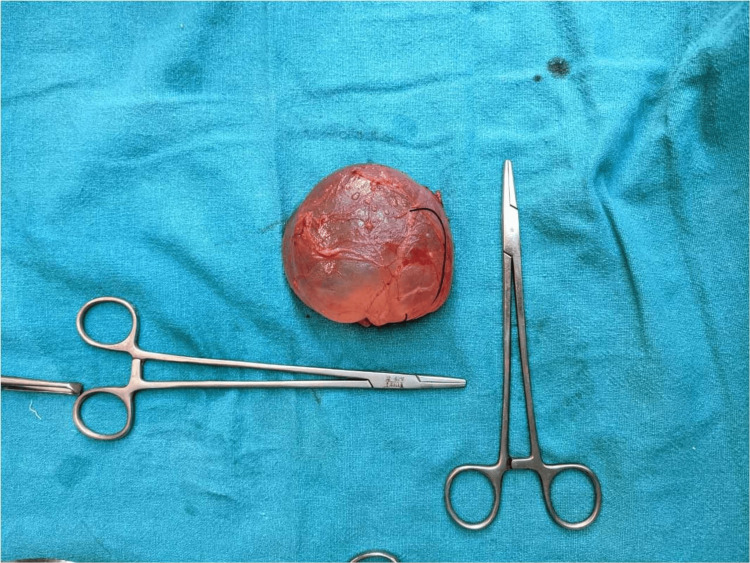
Completely excised mesenteric cyst

Intraoperatively, partial drainage of the mesenteric cyst content was performed, which reduced the volume and size of the mesenteric cyst. This made the mobilization of surrounding bowel loops easier and provided a clearer vision of the mesenteric cyst (Figures 3, 4).

There were several adhesions surrounding the cyst and no indications of ovarian or any other pelvic organ connection. The cystic growth was obviously entirely mesenteric. The time following surgery went without incident. On the 10th postoperative day, she was sent home with a histopathology report that suggested findings of reactive hepatocytes with laminated cyst wall and mixed reactive cellular infiltrates suggestive of a mesenteric cyst.

The patient was advised for a six-month follow-up with an ultrasonographic evaluation of the abdomen although the patient had no evidence of any recurrence or other pathology on the first two visits after being discharged.

## Discussion

Although mesentery cysts are frequently asymptomatic, they can cause symptoms due to compression of nearby structures, stretching of the mesentery as a result of the cyst's rapid growth, and rupture and infection. A mesentery cyst is typically described as one that extends into the retroperitoneal space, which includes a thick layer of endothelium or mesothelial cells. The mesenteric cyst can develop anywhere in the gastrointestinal tract's mesentery, from the duodenum to the rectum. Sixty percent of mesenteric cysts in a review series of 162 patients occurred in the small bowel mesentery, 24% in the large bowel mesentery, and 14% in the retroperitoneum. At the same time, it was uncertain in 1.5% of cases [6].

Single or more mesenteric cysts are not rare, and locularity inside the cyst is a common observation. The specific cause of the mesenteric cyst is yet unknown. Contributing reasons, according to some theories, include the lymph nodes' inability to interact with the lymphatic or venous systems or the lymphatics' obstruction as a result of trauma, infection, or tumors. When doing an autopsy on an eight-year-old, Italian anatomist Benevieni et al. first discovered them in 1507, and Tillaux et al. carried out the first successful surgical removal in 1880 [6,7]. Chylolymphatic, enterogenous, urogenital remnant cysts, simple (mesothelial), and teratomatous dermoid cysts are the five categories of mesenteric cysts, of which a simple (mesothelial) type of mesenteric cyst was seen in our case [4]. Chylolymphatic cysts are the most prevalent kind. They result from the lymphatic system's congenital misalignment. These are frequently isolated, unilocular, and have thin walls in the ileal mesentery. Enucleation can be performed without bowel resection since it has a separate blood supply. A diverticulum or duplication from the nearby colon causes enterogenous cysts. Since it has thick walls and draws blood from the nearby gut, removing the cyst invariably necessitates resecting the relevant segment of the intestine. Abdominal distension and nebulous pain are among the clinical signs in the advanced stages. Palpable masses, intestinal obstructions, and obstructive uropathies are possible in advanced stages [8]. Also, other surgical operations may discover cases by chance [8]. Additionally, it has been calculated that 3% of these cysts may develop a malignant transformation [9].

The differential diagnosis for this illness includes hydatid cysts, lymphangiomas, ovaries-related cysts, peritoneal cysts, and cystic teratomas, which can be differentiated by thorough history-taking, physical examination, and radiological investigations. A well-defined, fluid-filled cystic structure next to bowel loops may be visible on an ultrasound scan, frequently employed in the initial evaluation [8,9]. Enucleation of the mesenteric cyst, or the painless separation of the cyst from the surrounding mesentery leaves, is the recommended method [8,10,11]. A resection of the neighboring organ can be required if enucleation cannot be done safely because the cyst wall adheres to the mesenteric tissue and other tissues around it [12].

Deroofing, drainage, and partial cyst removal are other forms of treatment. These treatments have a higher propensity for recurrence and are, therefore, rarely recommended. Mesentery cysts are a relatively uncommon surgical ailment that requires hospital admission every 1/200,000 to 350,000 times [13]. Rokitansky gave the first accurate description of a chylous mesenteric cyst in 1842, and Tillaux carried out the first successful surgery for a cystic mass in the mesentery in 1880 [14]. Italian anatomist Benevenni first identified this condition by performing an autopsy on an eight-year-old boy in 1507 [15].

Hemorrhagic, serous, chylous, or contaminated fluid is only a few of the characteristics of the fluid that may be present inside the cyst. They can be extending from a few millimeters but they can occasionally be so big that they resemble tubercular ascites [16]. The benign growth of ectopic lymphatics in the mesentery without contact with the rest of the lymphatic system is the most widely accepted idea, put out by Gross [17,18]. Patients of any age may develop mesenteric cysts; children under the age of 15 account for about one-third of instances. The cyst may appear as an acute abdomen, as an incidental observation, or as an unspecific abdominal characteristic. Patients can present with lower abdominal pain and symptoms that are frequently associated with other abdominal conditions. They are frequently asymptomatic and discovered incidentally when patients are undergoing workup or receiving treatment for other conditions, such as appendicitis, small-bowel obstruction, or diverticulitis. Prakash et al. have reported a study depicting the frequency of symptoms that can be encountered in a case of a mesenteric cyst [4,19].

To arrive at a provisional diagnosis in cases of suspected mesenteric cysts, a thorough clinical examination, a complete history, all routine blood tests, and radiological investigations (X-ray abdomen erect, ultrasound abdomen, and computed tomography scan) are advised as the mainline investigations. The ultimate diagnosis must be histologically verified and can be made during laparotomy. Volvulus, leakage of infectious fluid, herniation of the colon into an abdominal defect, and blockage are secondary problems linked to mesenteric cysts [20]. The preferred method of treatment is still total excision, which prevents recurrence and potential malignant change. In some circumstances, bowel resection may be necessary if the intestinal structures are closely approximated by cysts or if blood vessels that supply the gut are involved. Less than 3% of cases result in malignant cysts [21]. Laparoscopic surgery has made it possible to avoid complete laparotomies. The cyst itself is removed using endobags. The patient can quickly return to work after a laparoscopic excision [22,23]. Regular follow-ups with an abdominal ultrasound are advised for such patients for early diagnosis of the recurrence of the mesenteric cyst and thereby reducing the morbidity. In case of any recurrence of the cyst, CT-guided aspiration is to be advised [24].

## Conclusions

The mesenteric cyst is a rare presentation that might have as few symptoms as short-term abdominal pain or discomfort or as serious symptoms and abnormalities that are consistent with an intestinal blockage. The patient needs to undergo a complete assessment. The partial cyst-draining technique may prove useful intraoperatively to make it easier to expose the operating field. There should always be an effort made to remove the entire mesenteric cyst. The cyst should subsequently be sent for histological and cytological analysis to determine the best course of treatment.
